# Non-Invasive Diagnostic Approaches for Kidney Disease: The Role of Electronic Nose Systems

**DOI:** 10.3390/s24196475

**Published:** 2024-10-08

**Authors:** Francesco Sansone, Alessandro Tonacci

**Affiliations:** Institute of Clinical Physiology, National Research Council of Italy (IFC-CNR), 56124 Pisa, Italy; francesco.sansone@cnr.it

**Keywords:** artificial intelligence, breath analysis, electronic nose, e-nose, GC-MS, kidney disease, pattern recognition, VOCs, volatile organic compounds, volatilomics

## Abstract

Kidney diseases are a group of conditions related to the functioning of kidneys, which are in turn unable to properly filter waste and excessive fluids from the blood, resulting in the presence of dangerous levels of electrolytes, fluids, and waste substances in the human body, possibly leading to significant health effects. At the same time, the toxins amassing in the organism can lead to significant changes in breath composition, resulting in halitosis with peculiar features like the popular ammonia breath. Starting from this evidence, scientists have started to work on systems that can detect the presence of kidney diseases using a minimally invasive approach, minimizing the burden to the individuals, albeit providing clinicians with useful information about the disease’s presence or its main related features. The electronic nose (e-nose) is one of such tools, and its applications in this specific domain represent the core of the present review, performed on articles published in the last 20 years on humans to stay updated with the latest technological advancements, and conducted under the PRISMA guidelines. This review focuses not only on the chemical and physical principles of detection of such compounds (mainly ammonia), but also on the most popular data processing approaches adopted by the research community (mainly those relying on Machine Learning), to draw exhaustive conclusions about the state of the art and to figure out possible cues for future developments in the field.

## 1. Introduction

**Figure 1:**
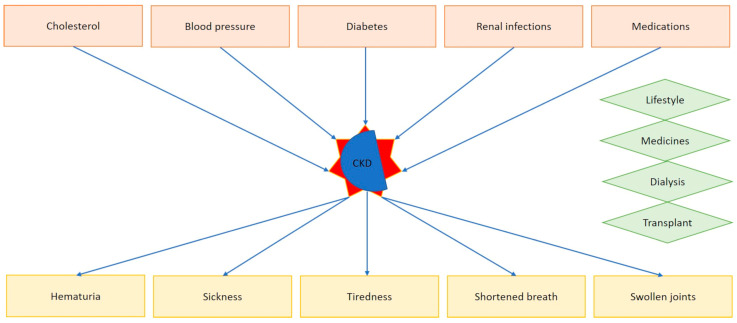
Causes (in red boxes), symptoms (yellow), and treatment opportunities (green) for CKD.

Kidneys are vital human organs whose main tasks relate to the filtering of fluids and waste out of the blood. A number of conditions are known to affect the kidneys, including those that impact their ability to clean the blood passing through them, with consequences for various entities at the organ level or even affecting other parts of the human body. Such conditions can lead to chronic kidney disease (CKD) or kidney failure [1]. CKD is a common condition, with an estimated prevalence of 13.4% globally [2], increasing with age and more prevalent in specific communities and ethnic groups, like African Americans and south Asians [3]. In their early stages, CKDs are mostly asymptomatic, whereas a handful symptoms can develop later, including hematuria (blood in the urine), sickness, tiredness, fatigue, shortened breath, and swollen joints, including hands, ankles, and feet. There are manifold possible causes for CKD occurrence, but the condition is often a combination of different health-related problems, including hypercholesterolemia, diabetes, hypertension, renal infections, or long-term usage of specific medicines, including lithium-based or non-steroidal anti-inflammatory drugs (NSAIDs). Actually, there is no cure to completely solve CKD in humans; however, its early detection, normally performed by urine or blood tests, can steer affected individuals towards the adoption of healthy lifestyles to delay complications associated with the disease. In more advanced stages, the detection of CKD occurrence can lead general practitioners and specialists to propose the patient tailored treatments, including medicine administration, dialysis, or, mostly in severe cases, kidney transplantation (see Figure 1).

Like other renal disorders, CKD, preventing blood from being properly cleaned, leads to the accumulation of toxins in the human body, resulting in marked differences in the chemical composition of body fluids, including blood, urine, and saliva. In fact, the occurrence and progression of the disease is normally associated with significant biochemical changes, and this is normally matched by important modifications to the volatile organic compound (VOC) patterns associated with exhaled breath composition [4]. Such occurrence can be exploited to design and implement innovative tools for monitoring the presence of CKD both at early stages—therefore, with diagnostic purposes—and also when the disorder is already diagnosed, with the aim of monitoring its progression. Recent technological advances in this regard are devoted to providing tools featuring minimal invasiveness for patients, reducing their burden and, at the same time, providing an affordable solution for healthcare providers. As such, electronic nose tools may represent a useful solution thanks to their adaptability, unobtrusiveness, and increasingly high performance, also taking advantages of the enhanced computational capabilities and results of modern artificial intelligence (AI) models [5,6]. Their deployment in various applications within the healthcare sector is already popular and is continuously gaining momentum; however, a systematic review of their use in the specific field of breath monitoring of individuals with CKD could represent a significant addition to current knowledge, possibly enabling the identification of strengths and existing gaps from technological and medical perspectives, therefore leading to useful cues for future developments in the field.

## 2. The Electronic Nose

### 2.1. The Historical Perspective: From the Ancient Age to the Modern Era

The tools nowadays known under the name of electronic nose (e-nose) represent a technological translation of the paradigm related to the biological sense of smell. Notably, the sense of smell is one of the five senses human beings use to perceive reality. It was already considered pretty important in ancient times, where the relationship between different kinds of body odors and pathological conditions was observed; however, due to the fact that human beings have evolved throughout the centuries towards a decrease in olfactory sensitivity, until some decades ago, together with the sense of taste, it was considered a neglected sense by clinicians and biomedical scientists [7], until its relationship with cognitive and neurological disorders was discovered (see [8] for an example). At that point, biomedical research on the importance and functioning of the sense of smell accelerated, also enabling important milestones across the fields of medicine and physiology, due to the discovery of mechanisms like the functioning of olfactory receptor cells, which turned out to be useful not only from a merely clinical perspective, but also in a different domain, namely, technological advancements.

**Figure 2:**
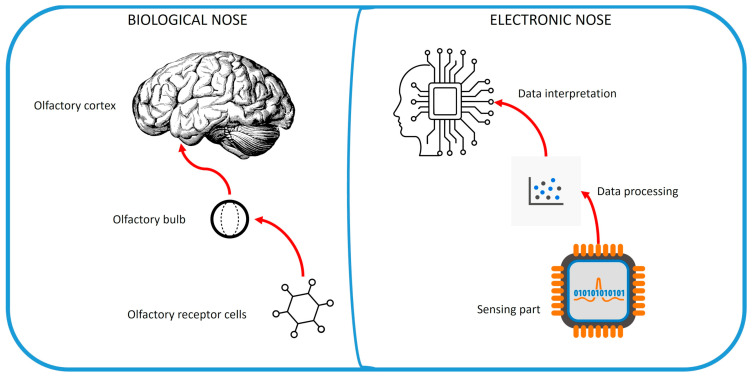
Analogies between biological and electronic noses.

Within this virtuous pathway of knowledge, in early 1980s, Persaud and Dodd, in the United Kingdom, conceived and realized the first “modern” e-nose tool. This precursor of actual e-nose systems included a sensing part, composed of three metal oxide sensors, and an intelligent portion, according to the knowledge of that time, and was initially capable of discriminating and identifying a remarkable set of odorants (up to 20) under controlled experimental conditions [9]. In the following years, various groups started working in the field, taking advantage of new sensing materials and algorithms for data processing and interpretation, with Gardner and Bartlett finally producing the term “electronic nose”, or e-nose, to link the approach to the biological counterpart related to the functioning of the sense of smell [10]. This has become better realized as, thanks to technological developments occurring year by year, the sensing part and the intelligent part of the system, composed of data processing and data interpretation, have been scaled up, each of them contributing to the full operativity of such a system. In such a framework, modern e-nose tools are made up of some key components, including a sensing part, represented by a fully functioning sensor array, of different sizes and characteristics, with different sensors usually presenting different selectivities for the various chemical compounds representing the target of analysis, and a technological part (usually a computer, or a similar, miniaturized device) for data storage and processing. Such a structure represents a straightforward parallelism with the biological sense of smell, where sensors are matched by olfactory receptors, placed at the olfactory mucosal level, and the computer represents the intermediate (olfactory bulb, matched by clustering methods) and upper (olfactory cortex, whose technological counterpart is represented by data interpretation models) parts of the extremely complex olfactory pathway using a merely reductionistic approach (see Figure 2) [11]. However, it should be still kept in mind that, despite significant technological advancements in the field, the performance gap between the biological and technological sense of smell is still largely present, due to space and technology constraints; however, leveraging on new techniques and methodologies, including those based on AI, would make this gap always less pronounced, until the systems could even match each other in terms of performances [12].

After a while, the first commercial devices, at first represented by the sensing part as a standalone solution, then going towards complete e-nose tools, started to appear on the market as soon as the early 1990s, mainly thanks to the AlphaMOS devices, followed by other competitors, somewhat promoting diversification, also in terms of the technology adopted for their development, some of which has arrived fairly recently. As such, as technological solutions appeared, a plethora of use cases started to enter the research and development market, including those for human breath analysis for the diagnosis of pathological conditions [13], the assessment of food quality [14], environmental monitoring concerning the presence of certain pollutants in air or water [15], as well as specific applications dealing with security and defense [16,17], highlighting the potential of e-nose systems in various fields of application.

### 2.2. The e-Nose Sensing Part: Principle of Operation

As outlined above, odor receptors, embedded within the olfactory mucosa, are matched, in their technological counterpart, by arrays of sensors, which are usually very sensitive, although poorly specific, to the volatile compounds of interest. Such devices should be capable of detecting very subtle traces of chemicals present in ambient air, similarly to what is performed by olfactory receptor cells in humans and animals [18], in turn delivering their signals throughout the olfactory pathway up to the olfactory bulb for “odotopic” characterization. The development of this part of e-nose systems should be performed taking into account some typical technological constraints due to power consumption, management of the whole system, and proper dimensioning of the related parts, which makes it impossible to fully replicate a natural sense of smell, composed of millions of olfactory receptor cells, which are mapped through arrays of sensors that typically number in the tens, if not even less. Such a constraint highlights the importance of the intelligent part of the e-nose system even more; pattern recognition algorithms and artificial intelligence (AI)-based models should guarantee high performance even in presence of a relatively limited number of signals and stimuli.

Throughout the decades and up to the present, the different technologies employed to realize the sensing part of the e-nose system include MOS sensors, which are probably the most widely used; metal–oxide–semiconductor field-effect transistor sensors, commonly known as MOSFET sensors; optic fiber sensors; solid electrolyte sensors; conductive organic polymers; and mass-sensitive sensors, each one of which features important positive characteristics and significant drawbacks (see Table 1 for a quick overview), which have determined their fortunes throughout the years, depending on the specific case study they have been applied to.

#### Novel Sensing Devices Eventually Applied to e-Nose Systems

As stated, the technological revolution we are currently experiencing has also extended to the field of sensors applicable to e-nose tools, as well as of related fabrication materials and principles. Therein, one of the most important milestones in the microfabrication domain is represented by developments occurring in the field of nanotechnology, including 2D materials like graphene, molybdenum disulfide (MoS_2_), layered molybdenum trioxide (MoO_3_) and stannic oxide (SnO_2_), and perovskite and phosphorene nanosheets, allowing for significant improvements in technology related to gas sensing [19,20,21,22,23,24]. Such materials are particularly suitable for e-nose applications due to their excellent efficiency and rapidity in energy transfer, biomolecule adsorption, and other interesting properties. Zero-dimensional options, including fullerenes, quantum dots, or nanoparticles, feature extremely high reaction speed, although their sensing capability can be negatively impacted by their tendency to aggregation due to weak electrostatic forces—including van der Waals interactions—they are submitted to, thus requiring their combination with other materials to overcome such limitations [25,26,27]. On the other hand, the main advantages of one-dimensional nanomaterials, such as nanowires, nanorods, nanotubes, and nanofibers, include the capability of carrying very high currents with reduced heating consequences, even if they are slower in terms of response and recovery times with respect to the existing alternatives [28,29]. Finally, three-dimensional materials, including hierarchical nanostructures, exhibit very fast responses and quick recovery times, with excellent mechanical properties with respect to the alternatives [30].

All the solutions presented above find their applicability within the scenario of e-nose systems, mainly concerning—for the domain investigated in the present review—the identification of VOCs in the framework of breath analysis, with 2D materials probably representing the most frequently used alternative in this specific domain [31].

### 2.3. The e-Nose Intelligent Part: Most Popular Models and Their Main Characteristics

The upper portion of the olfactory pathway runs from the olfactory bulb up to the olfactory cortex, which is in charge of translating the signals produced by the olfactory receptor cells into information, which leads to the identification and full characterization of the odorous compounds binding with the receptors and generating the signal. This part is technologically translated by pre-processing the signal produced by the sensor arrays, the setup and training of models and algorithms relying on AI, and similar principles. Overall, among the number of models available nowadays, it is deemed tricky develop specific solutions, which are more suitable for e-nose applications. However, usual choices for such a framework include Principal Component Analysis (PCA) [32], Support Vector Machine (SVM) [33], Artificial Neural Networks (ANNs) [34], and Linear Discriminant Analysis (LDA) [35], among others.

#### 2.3.1. Principal Component Analysis

Principal Component Analysis, commonly known as PCA, is a method usually applied to reduce the dimensionality (and the complexity) of some datasets, which are often characterized by huge quantity of data and significant intrinsic complexity. PCA takes as input a large set of variables and transforms it into a smaller dataset still containing most of the information stored in the original dataset. The main challenge that PCA faces relates to the optimal management of the trade-off between dimensionality (and complexity) reduction and accuracy (and informativity) maintenance. Thus, its main idea is quite simple, that is, to reduce the number of variables to be analyzed within a dataset as much as possible without losing the original information in this transformation.

Like any other model, PCA also comes with advantages that have helped the model in its fortunes across the years, and drawbacks, which in turn have hampered its further exploitation. Advantages include the possibility to visualize data through dimensionality reduction, the possibility to remove multicollinearity and noise from data, and the reduction in model parameters and training time. On the other hand, it still requires fairly significant run times, and it presents important problems concerning feature interpretability, as well as a significant loss of information during transformation, especially in specific use cases. Furthermore, it only makes use of linear dimensionality reduction, and it is still quite heavily affected by data outliers.

The usage of PCA in the framework of e-nose tools is quite frequent, and many authors have demonstrated its applicability in this specific use case scenario, leveraging its light weight and extreme simplicity (see [36,37,38] for some examples).

#### 2.3.2. Support Vector Machine

Support Vector Machines, popularly known as SVMs [39], include a set of supervised learning methods, commonly used for classification and regression purposes, as well as for the detection of outliers within a dataset. SVMs are capable of performing linear as well as non-linear classification using the kernel trick, in turn allowing for the representation of data through pairwise similarity comparisons between the original data points by applying a kernel function, transforming such data into coordinates within higher dimensional feature space. SVMs feature a number of advantages, including their excellent effectiveness in high-dimensional spaces, as well as in cases where the number of dimensions is higher than the number of samples. They are very memory-efficient, as they use a subset of training points in the decision function, and they are also quite versatile, with different kernel functions that can be implemented regarding decision functions. On the other hand, the main drawbacks of SVMs include their inability to directly provide probability estimates, in turn being calculated through a tricky, expensive approach, and their tendency to overfit when the choice for kernel functions and regularization term is not optimal.

Concerning e-nose solutions, SVMs have been used in several research articles in the field, taking advantage of their powerful capabilities, which made them the best choice in many use cases before the onset of Deep Learning models in the past decade. E-nose tools using an SVM reported optimal performance in various scenarios, including the classification of cardiovascular conditions [40], the detection of a number of different diseases through urinary volatile analysis [41], and the identification of contaminants in indoor air [42].

#### 2.3.3. Artificial Neural Networks

Artificial Neural Networks, also known as ANNs or Neural Networks, include a technological approach based on the functioning of the human brain and the nervous system, as they simulate the electrical activity of the brain portions, in particular of the neurons. As such, processing elements are connected to each other, and arranged into layers, with the output of one layer representing the input of the subsequent layer and so on. Then, information is passed from one layer to another, with some connections being strengthened and others being weakened depending on weights assigned from time to time, similarly to what occurs within the human brain’s synapses.

The use of ANNs should be considered taking into account the several benefits and limitations they deliver. Among them, they show an excellent ability to work in parallel, enabling large quantity of data to be processed simultaneously; furthermore, they are quite tolerant to noisy data and can be easily updated in case new data are included in the dataset. Finally, they perform quite well in the presence of very specific and complex problems. However, ANNs do come with significant limitations, too. The limited interpretability of the outputs, especially when trying to analyze the intermediate layers, is one of the main issues they present. Then, the computational process is often quite burdensome, especially in the presence of multiple iterations; therefore, it is deemed poorly applicable to specific use cases where computational load can be considered a constraint.

Among the main usages of ANNs in the framework of e-nose systems, the most widely used models include Multilayer Perceptron (MLP), employed for VOC assessment [43], food-related studies [44], environmental monitoring [45], and security tasks [46]; Extreme Learning Machines (ELMs), finding use in VOC assessment [47] and environmental use cases [48]; Convolutional Neural Networks (CNNs), also employed in food assessment [47] and VOC determination [49]; as well as other models, like Long-Short Term Memory (LSTM) [50].

#### 2.3.4. Linear Discriminant Analysis

Linear Discriminant Analysis, abbreviated as LDA, is a very popular discriminant technique, as it is capable of optimizing the variance between and within classes by transforming the original variables through linear combinations to ultimately ensure an effective separation of classes, in order to recode original datasets into spaces of lower dimensions, highlighting the distinction of classes and decreasing the overall computational complexity. Differently from PCA, LDA represents a supervised model, as it employs a training dataset to build up a model for assessing new data against the model established. Even for this approach, we can enumerate a number of advantages and limitations—among the first ones, its simplicity and portability, even taking into account its fairly good performance. On the other hand, it requires important assumptions on the features, including their normal distribution, and it turns out to not always be good for a limited number of categorical variables or in the presence of small datasets.

Concerning e-nose systems, LDA was successfully employed—for example, in the food industry [51,52], as well as for healthcare purposes—in breath analysis [53].

#### 2.3.5. k-Nearest Neighbors

k-Nearest Neighbors, popularly known as kNN, is a very popular, non-parametric supervised learning method, used both for classification and regression purposes, where inputs are represented by the k closest training examples in a dataset, whereas the outputs are class memberships for classification and property values for the object in regression kNN systems. Such models are straightforward and easy to train, although their significant sensitivity to the local structure of the data represents one of their main limitations.

#### 2.3.6. Random Forest

Random Forest is a popular Machine Learning method suitable for both classification and regression tasks, operating by building up a number of decision trees at the training phase. In classification tasks, its output is represented by the class selected by the most trees, whereas in regression tasks, the output is represented by the mean prediction of the individual trees. Despite being viewed as a black box, it provides several advantages, among which is relative stability with respect to overfitting.

To enhance the readability of this section, the main models and their advantages and drawbacks are summarized in Table 2.

#### 2.3.7. Novel AI Models to Be Applied to e-Nose Systems

Beyond the typical AI models, mainly based upon Machine Learning algorithms, as abovementioned, recent e-nose tools have started using approaches that represent the current state of the art when it comes to the use of AI, relying on Deep Learning (DL) principles. In this regard, Convolutional Neural Networks (CNNs) are commonly used, with fairly good performance—yet this depends on the specific use case—compared to applications where Machine Learning counterparts are commonplace [54,55]. Other approaches, like those using more complex models, including the adaptive convolutional kernel channel attention network (AKCA-Net), have been employed [56], presenting excellent performance, yet being superior to other models, like CNNs or Efficient Channel Attention (ECA), among others, however with significant burden when it comes to the computational costs that should be taken into account when selecting the most suitable solution for a given application. To partially cope with that, some researches have also used Transfer Learning (TL) approaches, with the aim of reducing the computational costs associated with the full training of a complex DL model, merging together the significant advantages of DL with a reduced need to perform the whole learning chain for the system, thus overall saving time, resources, and also diminishing the associated carbon footprint [57,58,59].

### 2.4. Validation: A Crucial Step for e-Nose Systems

In general terms, when e-nose systems are developed and applied to a given use case scenario, both in environmental monitoring and in health conditions detection, it is of utmost importance to foresee a validation process of the instrument and of the whole analysis pipeline in order to avoid false discoveries by the tool and misinterpretation of the results by the operator. As such, during the sampling process, sensor malfunctions could occur, either reporting incorrect values or stopping their functioning for a short amount of time, in turn possibly representing a significant criticism of the whole process. For this reason, it is necessary to adopt solutions that are fast, computationally efficient, and reliable, thus enabling eventual action, including the possibility to resample and recalibrate the models or to check the eventual failure of one or more sensors within the e-nose tool.

This occurrence could be applied to all the use cases where e-nose systems are employed, including environmental monitoring [60], the food industry, as well as healthcare, where it is possible that the criticisms eventually occurring are even more subtle and, therefore, to be faced in a timely manner.

Among the most relevant works to date, Mirshahi and colleagues proposed an algorithm that could fit the problem well, with fairly good performance, also when it comes to the real time validation, with low computational burden and easy implementation, therefore reducing possible issues related to the eventual delay in recognizing sensor failure and related issues [61]. However, it is worth noting that validation, together with standardization, still represents one of the most important limitations e-nose systems experience, especially when it comes to their clinical translation and deployment.

## 3. Materials and Methods

A systematic literature review was conducted, according to the PRISMA guidelines [62], on several databases, including PubMed, ScienceDirect, and Google Scholar, according to the following terms: ((“E-nose” OR “Electronic nose” OR “Olfaction technology” OR “Odor detection”) AND (“Kidney” OR “Chronic Kidney Disease” OR “Chronic Kidney Disorder” OR “Kidney Failure” OR “Renal Failure”)).

The records included were related to research on humans published between 1 January 2004 and 31 July 2024 in the English language—excluding systematic reviews, meta-analyses, and case report studies—that could possibly be employed to foster discussion or to cross-search further related articles. The time scale adopted was chosen to ensure the inclusion of up-to-date technologies and techniques in terms of advancements, particularly within e-nose devices and related AI models.

Each article was analyzed in terms of disease investigated, technology employed, and overall performance, including sensitivity and specificity or accuracy.

## 4. Biomarkers of Chronic Kidney Disease

**Figure 3:**
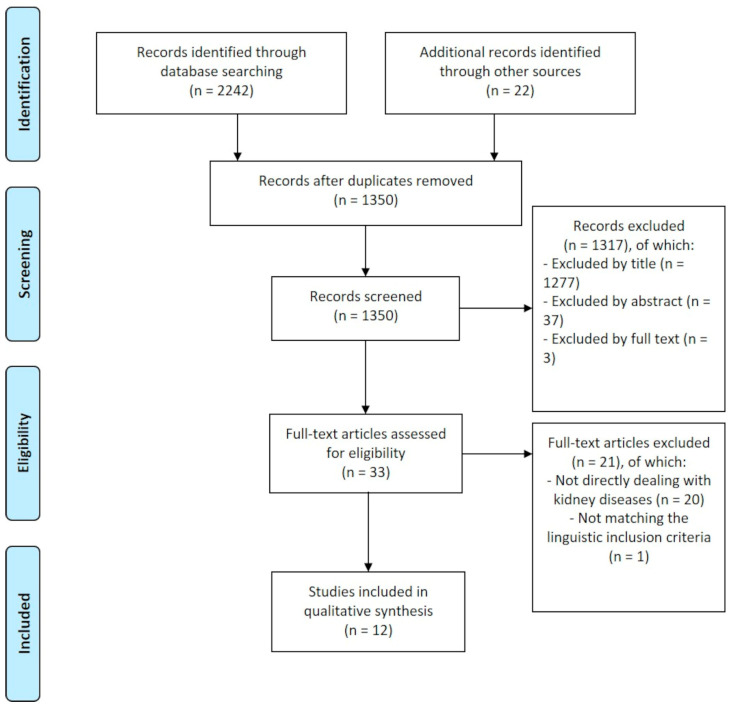
The PRISMA flowchart.

The results of the literature search are graphically displayed in Figure 3. After duplicate removal, 1350 records were screened, 1317 of which were excluded. The vast majority of them were excluded by title (n = 1277), n = 37 by abstract, and the remaining n = 3 by full text. n = 33 papers were assessed for eligibility, 12 of which were finally included in the qualitative synthesis of the present review. According to the papers retrieved, the abnormal concentration of a number of compounds appears to be present in biological samples drawn from individuals suffering from kidney disease, due to the accumulation of toxins in their body, in turn caused by kidney malfunction.

More specifically, an increased concentration of several substances, like ammonia and isoprene nitrogen-containing compounds, including isoprene, aldehydes, and uremic toxins, was observed in nephropathic subjects, whereas methylamines showed contradictory results (Table 3) [63,64,65,66,67].

Nitrogen-containing compounds, including ammonia and di- and tri-methylamine, are mostly increased in their concentrations regarding renal conditions. According to a source in the literature [82], they are among the main responsible compounds for the peculiar breath odor of patients with renal failure, including those with uremia, resembling a typical urine smell [83]. As such, ammonia comes from the metabolism of proteins and nucleic acids, it is converted to urea and ammonium salts, and is then eliminated from the human body through urine, this process, however, being impaired in those with a significant urea imbalance possibly due to kidney failure, where urea concentration in body fluids is abnormally high [64], or similar conditions. In terms of concentrations in breath samples, Romani and colleagues [66], using selected ion flow tube mass spectrometry (SIFT-MS), found that a subject presenting an ammonia concentration equal to or above 6450 ppbv has a probability of being affected by CKD, equal to 75%, with just 0.01% probability to be misclassified as having CKD, highlighting the excellent predictive value of the compound in the framework of renal conditions. Ammonia was also found increased among children and adolescents with CKD (284 vs. 556 ppbV on average) with respect to healthy counterparts [67]. However, when it specifically comes to methylamine, in pediatric patients with CKD, it was found to be lower than among healthy individuals (6.5 vs. 10.1 ppbV) [67], raising uncertainty about the effective validity of this biomarker for CKD, at least in this specific cohort.

Also, Demirjian and colleagues [65] retrieved interesting results about acetone. Acetone is present in the breath of individuals due to a plethora of conditions, including diabetes mellitus and ketonemia, but is also one of the major volatile organic compounds (VOCs) present in human breath. It is mainly produced by hepatocytes through the decarboxylation of acetyl-CoA in excess, derived from the β-oxidation of fatty acids, and formed by the decarboxylation of acetoacetate, in turn derived from lipolysis or lipid peroxidation [80,84]. Overall, it is linked to the presence of ketone bodies, which are formed when the human body, in need of energy, employs fats instead of glucose. According to Romani and co-authors [66], acetone can be used to distinguish individuals with renal failure from those not affected by the condition, with a threshold value of 345 ppbv, granting probability for those who present with an acetone concentration in the breath of 88.6% (or higher) to be affected by CKD, but still presenting a false positive likelihood of 54.2%, thus decreasing the overall predictive value of the acetone assessment for CKD.

According to the work by Demirjian and colleagues [65], acetaldehyde was highly prevalent in breath samples of individuals with renal failure with respect to healthy controls (81 (61–136) vs. 28 (18–41) ppb, *p* < 0.001) and, in any case, it should remain below 80 ppb, not exceeding this level too much even after the ingestion of ethanol, which represents its progenitor compound [85,86,87]. Commonly, acetaldehyde is related to oxidative stress and inflammatory mechanisms; it is capable of binding to the free amino acid groups of several proteins, also modifying the metabolism of apolipoprotein and fatty acids [88,89]. Its relationship with the inflammatory mechanisms and oxidative stress reported above makes it a key predictor of various diseases, including CKD.

The same group [65] reported a significant increase of 2-propanol in breath samples of those with kidney failure, with values around 219 (172–328) ppb versus 31 (26–38) ppb (*p* < 0.001) reported in healthy individuals. This VOC originated due to the reduction of acetone, and was reportedly abnormal in the exhaled breath of patients affected by lung cancer and liver diseases, as well as in the breath condensate of those with cystic fibrosis and chronic obstructive pulmonary disease (COPD) [90,91,92,93].

Isoprene was also abnormally elevated in breath samples of pediatric patients with CKD, with values reaching around 70.5 ppbV on average, largely higher than the concentrations reported in healthy counterparts (49.6 ppbV on average) [67]. Isoprene is one of the most common VOCs in human breath, being formed along the mevalonic pathway of the synthesis of cholesterol in the cytosolic fraction [80]. This important compound was found as a biomarker of some conditions relating to cholesterol metabolism, including hypercholesterolemia, with its concentrations being lower in children and progressively increasing up to 25 years of age [84], although its relationship with specific metabolic pathways in kidney conditions is still poorly clear.

Pentanal and heptanal were also found to be higher in pediatric patients with kidney failure, markedly impacting their breath profile, especially in more severe stages of CKD [67], with values exceeding 9.3 ppbV and 5.4 ppbV for patients, versus 5.30 ppbV and 2.78 ppbV for controls, respectively. Those compounds, also retrieved in biological fluids of patients with completely different conditions, including lung cancer, are normally considered as reliable markers for oxidative stress, which turns out to be correlated to kidney diseases, rather than directly to a kidney damage by itself. Such abnormalities were mainly observed in patients receiving a transplant, arguably due, at least partially, to immunosuppressive treatments [94], whereas their relationship with the pathophysiology of renal diseases in patients with milder conditions is still under investigation [95,96].

Exhaled nitric oxide (NO) was also seen as abnormally concentrated in breath samples of those with glomerulonephritis, with levels around 29.5 ± 1.4 vs. 18.7 ± 1.0 ppb, *p* < 0.0001, and higher exhaled NO output (166.6 ± 6.8 vs. 95.5 ± 5.6 nl/min/m^2^, *p* < 0.0001). Similarly, plasma NO2-/NO3- concentrations were also higher in the same individuals [81].

Ethanol was also seen to be nearly doubled in breath samples drawn from pediatric patients with CKD (146 vs. 82.4 ppbV on average) [67], differing from controls either in those with mild or moderate disease; however, this was possibly related to the contamination of the external environment, especially when it comes to inpatients [67].

Finally, dimethyl sulfide (DMS), a potential marker of oxidative stress, was retrieved as being abnormally present in biological samples of patients with CKD when comorbid for diabetes [97]. However, this compound is not included in Table 1 due to its presence having only been reported in individuals who are comorbid with diabetes, as stated above.

## 5. Electronic Nose Tools and Chronic Kidney Disease

Table 4 outlines the main studies dealing with the development and testing of electronic nose-like tools applied within the framework of CKD.

Most studies have employed such devices to characterize exhaled breath of individuals with CKD [99,100,101,102,103,104,106,107,109], followed by urine samples [103,105,108]. However, one of the seminal works in this regard, that by Voss and colleagues [98], focused on the characterization of body odors via the application of an air sampling tool linked to three thick-film metal oxide-based gas sensors and to a miniaturized computer interface to be placed on the leg of patients with chronic and end-stage renal diseases. Through the analysis of principal odor components (POCs), the approach allowed for the scientists to distinguish between healthy and diseased individuals with 100% accuracy, whereas discrimination between patients with chronic and end-stage renal disease was performed with 95.2% accuracy when using the two first POCs and increased to 98.4% when the third POC was also considered.

MOS sensors are the most popular, also when it comes to e-nose devices used or developed for breath analysis in the framework of renal diseases, as also happened with other conditions, like, for example, cancer (see [110] for a systematic review). This is mainly due to their lower costs with respect to alternative solutions on the market, making them ideal for point-of-care devices with requirements including fast response time, fair accuracy, and affordability. On the other hand, they suffer from fluctuations due to environmental conditions and drifts; therefore, they should be well calibrated and employed in controlled environments [29]. In this regard, Guo and collaborators [99] used a sensor array composed of 12 sensors—in turn sensitive to H_2_, CO, VOCs, H_2_S, CO_2_, NH_3_, NO, and NO_2_—set in a 600 mL stainless steel chamber to analyze the breath samples of volunteers in one of two different ways depending on the experimental demands, including the dead-space air from the upper airway or alveolar air from the lungs. The study population was composed of 110 subjects with renal conditions, 110 with airway inflammation, 117 with diabetes, and 108 healthy controls. After signal pre-processing and feature extraction by means of Principal Component Analysis (PCA), the resulting data were classified according to a k-Nearest Neighbor (kNN) approach. Relating to the present review’s focus on renal diseases, the results obtained by Guo with respect to that specific cohort reported a sensitivity of 86.57% and a specificity of 83.47% for those with renal failure, values close to those obtained on diabetic individuals, and much higher than those reported for patients affected by airway inflammation.

Some years later, Jayasree and colleagues [102], using a 500 mL Tedlar bag, collected breath samples from a cohort of 40 individuals (30 males; 10 females) affected by CKD, analyzing them through a tool composed of TGS2444, MQ135, MQ137, and TGS826 sensors. After feature extraction, a Support Vector Machine (SVM) was applied to distinguish between pre- and post-dialysis subjects, allowing for correct classification according to the outputs of the TGS2444 sensor (the best performing one) in 83% of cases using two features, increasing to 88% of cases when applying three features, providing overall satisfying results within the specific use case scenario.

Saidi and collaborators [103] investigated the exhaled breath of volunteers with CKD, diabetes mellitus, and healthy individuals using an e-nose and gas chromatography–quadrupole time-of-flight mass spectrometry (GC/Q-TOF-MS). More specifically, the e-nose system included an array of six commercial chemical gas sensors (MQ-2, MQ-3, MQ-9, MQ-135, MQ-137, and MQ-138), manufactured by Hanwei Electronics Co. Ltd., Zhengzhou, China. The volunteer cohort was composed of 16 patients with CKD, 6 with diabetes mellitus, and 22 controls (total of 44 individuals, of which 30 were male and 14 were female), from which an overall 264 breath samples were collected (216 samples from 36 subjects to train the model; 48 samples from 8 subjects to test the model). The exhaled breath for all participants was collected into a 1 l Tedlar bag. After feature extraction, including the conductance slope, the area under the curve, and the conductance change due to breath exposure for the various sensors, pattern recognition methods were implemented, including HCA, PCA, and SVM. According to the authors, 100% of samples were correctly classified by the SVM, possibly due to the overfitting tendency within a relatively small quantity of data to build the model, even if a leave-one-out cross-validation approach was applied to avoid, or reduce, the problem of overfitting.

A work by Kalidoss and colleagues [107] further highlighted the role of MOS sensors to characterize the breath samples of individuals with CKD. Here, an MQ-135 device (Figaro Engineering, Inc., Rolling Meadows, IL, USA), extremely sensitive to ammonia, was placed within a sampling system made up of a borosilicate glass measurement chamber, featuring a volume of 500 mL to accommodate the complete alveolar breath. Fifty-one individuals with CKD and forty-seven healthy controls were included in the study, performed according to a feature selection step, followed by PCA and SVM and kNN models for fine classification between cohorts. Using the Information Gain Feature Selection (IG FS) approach, the authors achieved a significant accuracy value of 85.7% for SVM and 83.6% for kNN classification models, confirming the applicability of e-nose systems based on MOS sensors to solve the specific problem.

Chan and co-authors [106], with a similar approach, based on a vertical-channel organic semiconductor (V-OSC) sensor, tried to discriminate between CKD patients depending on their disease stage, using data from 19 stage 1, 26 stage 2, 38 stage 3, 21 stage 4, and 17 stage 5 patients, for an overall 121 individuals affected. The exhaled breath samples were collected in a 500 mL plastic bag, then transferred into a second bag through a desiccation cylinder, and finally connected to the inlet of the gas measurement system. Classical statistics were employed for data analysis, revealing an ROC AUC of 0.835 across CKD at first and at any stage at 974 ppb (sensitivity: 69%; specificity: 95%).

Using a different technological approach, Marom and co-authors [100] applied two to three gold nanoparticle (GNP) sensors, cross-reactive chemiresistors based on four types of spherical GNPs with a core diameter of 3–4 nm, featuring organic ligands, including 2-ethylhexanethiol, tert-dodecanethiol, hexanethiol, and dibutyl disulfide. The signal collected from CKD patients (n = 6 with stage 2, n = 16 with stage 3, n = 12 with stage 4, n = 8 with stage 5 CKD, and n = 20 healthy controls) were analyzed using an SVM. According to the authors, 79% accuracy was reached between early-stage CKD and controls, whereas stage 4 and stage 5 CKDs were distinguished in 85% of cases. A slightly worse result was otherwise obtained between early and advanced CKD, discriminated in 76% of cases.

Later on, the same group [101] developed a protocol to analyze breath samples from patients with end-stage renal disease to study the impact of hemodialysis on their volatiles. In the study, the authors selected a group of sensors from an array of chemiresistors based on different nanomaterials, including organically functionalized GNPs and random networks of single-walled carbon nanotubes (SWCNTs) capped with organic films. Twenty-six patients and eleven healthy controls were analyzed in the investigation, with Discriminant Function Analysis (DFA) applied to select sensors and related features for discrimination between the two groups. Finally, training and test subsets were created and evaluated in terms of performance, with the test set attaining 80% correct discrimination between patients and controls.

Le Maout and co-authors [104] developed an e-nose system with an array of 11 polyaniline nanocomposites sensors to be employed to analyze seven cycles of 51 injections of different concentrations of ammonia, a known biomarker for CKD in exhaled breath and other biological fluids. After feature extraction, a number of models were employed for data analysis, including Linear Discriminant Analysis (LDA), Random Forest (RF), Support Vector Machine (SVM), and Multilayer Perceptron (MLP). With Recurrent Feature Elimination (RFE), the performances obtained with the different models were 91% for SVM, 87% for MLP, and 84% for LDA, at the same time allowing for a reduction in the number of sensors employed down to eight, six, and four, respectively.

Finally, Li and colleagues [109] developed an Fe_2_Mo_3_O_8_/MoO_2_@MoS_2_ nanocomposite sensor to detect ammonia in the exhaled breath of patients with early- and late-stage renal diseases (three for each of the two groups) against three healthy controls. According to the authors, the device was capable of detecting ammonia concentrations with R^2^ = 0.99 using the sensors’ outputs, demonstrating their potential applicability for the breath characterization of individuals with CKD at various stages.

Aside from investigations around breath, urine was also considered in a couple of studies using e-nose-like systems within the framework of kidney diseases. CKD was investigated by Jokiniitty and colleagues [105] concerning the analysis of urine headspace samples with a tool based on field asymmetric ion mobility spectrometry (FAIMS). Ninety-five patients with different degrees of CKD, according to their Glomerular Filtration Rate (GFR) class, entered the study, their extreme being discriminated with an accuracy of 81.4% using classical statistical analysis.

An investigation around renal cancer was performed by Costantini and collaborators [108] using urine headspace samples from 252 individuals, of which 110 were renal patients and 142 healthy controls, analyzed through the commercial Cyranose 320^®^ (Smith Detections, Pasadena, CA, USA), composed of an array of 32 sensors with different selectivities towards a set of VOCs. After PCA, the authors were capable of discriminating the two groups with a specificity of 89.4%, a sensitivity of 71.8%, and featuring a positive and negative predictive value of 84.04% and 80.37%, respectively, highlighting the potentialities of this approach for renal cancer detection from urine samples.

## 6. Discussion

CKD represents one of the most burdensome clinical conditions worldwide, with an estimated 10% of the global population expected to be affected by the disease [111], which is roughly 850 million people, mostly living in low- and lower-middle-income countries, however with a poor awareness of the disease at the population level [112]. This fact prevents the scientific and clinical community from undertaking large population studies, especially in areas globally where the need for carrying out such investigations is higher. This phenomenon is reflected in the relatively poor presence of related studies in the scientific literature, especially when it comes to new technologies and approaches to the problem, including the application of electronic noses (and similar) tools for investigating, in a minimally invasive way, the presence of biomarkers in biological fluids, including breath, urine, or saliva. Such deficit is particularly true when compared with other domains of the clinical field, like cancer [110], diabetes [113], or heart failure [114], to make some examples of conditions where different approaches with respect to traditional ones are commonly adopted. However, despite the relative scarcity of papers dealing with the topic, the present review highlighted that the main application of e-nose systems within the framework of CKD is represented by exhaled breath analyses, which are prevalent in the literature with respect to other biological fluids, including urine, in turn mostly considered considering its headspace. It is also quite common for other conditions—as reported, for example, in different kinds of cancer [110,115], where exhaled breath is preferred due to the information provided on cancers and their intrinsic nature—to be properly analyzed through e-nose tools, in turn being capable of dealing with gaseous samples. Focusing specifically on the type of electronic nose systems employed to investigate using exhaled breath from individuals with CKD, it came to our attention that the most popular technology is represented by metal–oxide sensors, the so-called MOS sensors, followed by nanocomposite- or nanomaterial-based devices. In fact, MOS sensors offer a wide range of advantages, which are well suited to the application of e-nose principles in the reference domains where it is expected to take part, including for biological fluids assessment. Indeed, MOS sensors are low-cost, quite sensitive, and offer a quick response, with significant environmental sustainability, afforded by their relatively long life (up to 5–10 years) and low power consumption. On the other hand, they are sensitive to environmental conditions; therefore, efficient control in terms of temperature and humidity should be guaranteed, also as a check for drifts and cross-sensitivity to gases other than those targeted. However, e-nose systems, also relying on signal conditioning and processing, as well as approaches for information extraction from raw data, including PCA, Artificial Neural Networks, and more, represent the ideal playground for such sensors, minimizing their possible drawbacks and maximizing their potentialities. At the same time, conversely to the scientific literature dealing with other disorders [110], possibly due to the relatively low number of articles about e-nose use within a kidney disorder framework, commercial e-nose devices are poorly used in this specific field, with just one research work [108] using the Cyranose^®^320 device (Smith Detections, Pasadena, CA, USA), one of the most popular solutions on the market [108], to analyze urine headspace samples within a renal cancer framework.

However, regardless of the approach adopted, it is evident from the current literature review that most of the studies published to date report optimal performance (generally above 80%) in the task of discriminating between VOCs emitted in biological fluids of individuals with kidney disorders and controls, with such results also obtained in many cases when attempting to distinguish between patients with different stages of kidney conditions, a task significantly more difficult than that of discriminating healthy and diseased individuals, thus enhancing the importance of e-nose devices within a minimally invasive biological fluid analysis framework in the clinical and (bio-)medical fields.

## 7. Conclusions and Future Perspectives

Research around biomarkers for the detection of kidney diseases is continuously growing and takes advantage of technological developments in various fields, including the specific area of e-nose systems. In this regard, both the discovery of novel, more efficient, and cost-effective sensing materials and the development of new methods, based on AI, for enhancing the capabilities of e-nose tools to recognize specific patterns related to the presence or absence of a given disorder, have the capability to promote the scaling up of solutions relying on e-nose systems and similar approaches. In fact, it is common belief that such technological advancement will allow for more frequent adoption of e-nose systems within the framework of renal disease detection and monitoring, within the “p4 medicine” scenario, and it is also expectable to have continuously enhanced performance from such solutions as long as they are developed and commonly used in the specific field of investigation, thanks to the continuous learning and training from experience of the intelligent part of e-nose tools. From the point of view of sensing materials, it is expectable that novel solutions, both relying on new nanomaterials and on the combination between different classes of existing materials, are expected to be released and used in the e-nose tools eventually developed for various purposes, including healthcare and kidney disease diagnosis support and monitoring. Concerning AI, hybrid solutions with DL and TL will probably replace, at least partially, actual Machine Learning approaches, which still play the main role in contemporary e-nose tools.

From the development and release of large datasets, from an open science perspective, with the continuously larger adoption of AI solutions, further benefits in this regard are expected, with potential breakthroughs for both clinicians, in terms of diagnosis accuracy, and patients. However, technological advancements will be fruitful for clinical practice with a merely uniform, universally accepted validation pathway, in turn ensuring the full deployment of such solutions in the related scenario, ultimately leading to an effective benefit for the life quality of patients and their caregivers, and significant monetary savings for healthcare providers.

## Figures and Tables

**Table 1:** Advantages and drawbacks of the most popular e-nose sensing approaches (MOSFET: metal–oxide–semiconductor field-effect transistor; MOS: metal–oxide sensor).

Technology	Pros	Cons
MOS	Low-cost, effectiveness, portability, reliability	Sensitivity to environmental conditions (temperature, humidity), baseline drifts, somewhat limited lifespan
MOSFET	High efficiency in power transform implementation, easy fabrication and integration, miniaturization	Heat generation, easy to be damaged
Optic fiber sensors	High sensitivity and accuracy, immunity to electromagnetic interference, small size and light weight, multiplexing capability	More expensive than alternatives, quite fragile, need maintenance
Solid electrolyte sensors	Safety, thermal stability, wide electrochemical window, good cycle performance	High interface impedance, air sensitivity
Conductive organic polymers	High conductivity, excellent electrical stimulus, biocompatibility and biodegradability	Suboptimal durability
Mass-sensitive sensors	Stable sensing materials, simple and affordable fabrication, rapidity of measurement	Sensitive to external disturbance, poor selectivity

**Table 2:** Advantages and drawbacks of the most popular intelligent models applied to e-nose systems (ANNs: Artificial Neural Networks; kNN: k-Nearest Neighbors; LDA: Linear Discriminant Analysis; PCA: Principal Component Analysis; RF: Random Forest; SVM: Support Vector Machine).

Model (s)	Pros	Cons
PCA	Easy data visualization; multicollinearity and noise removal; reduced training time and model parameters	Significant run-times; limited features interpretability; significant loss of information during data processing; affected by outliers
SVM	Effective in high dimensional spaces; good memory efficiency; good versatility	Unable to provide direct probability estimates; tendency to overfit
ANN	Parallel operation; tolerance to noisy data; easily updated with new data; good performances in complex problems	Limited output interpretability; burden of computational process
LDA	Simple; portable; good performance	Normal features distribution required; not optimal with small dataset or few categories; tendency to overfit
kNN	Lack of training period: simple to implement	Not optimal with large dataset and with high dimensional data; sensitivity to noisy and missing data
RF	High accuracy; robustness to noise; handling missing values and numerical and categorical data; somewhat stable to overfitting	Poorly interpretable; high computational costs and memory usage

**Table 3:** Altered biomarkers in clinical conditions related to kidney disorders and their physiological sources (CKD: chronic kidney disease).

Clinical Condition (s)	Altered Biomarker (s)	Physiological Source (s)	Alteration Type	Reference (s)
Uremia, kidney impairment, CKD	Nitrogen-containing compounds (ammonia, dimethylamine, trimethylamine)	Protein metabolism [68]	Mostly increased	[63,66,67,69]
CKD, renal failure	Ammonia	Protein metabolism [65], blood urea [70,71,72,73]	Increased	[63,64,65]
Renal failure	2-propanol	Acetone reduction [65]	Increased	[65]
Renal failure	Acetaldehyde	Oxidative stress, inflammatory processes [74,75,76,77,78]	Increased	[65]
CKD	Acetone	Decarboxylation of acetoacetate and acetyl-CoA [63], fatty acid metabolism [79]	Increased	[66,69]
CKD	Isoprene	Cholesterol metabolism [80]	Increased	[63,67]
Chronic glomerulonephritis	Nitric oxide	Metabolic processes	Increased	[81]
CKD (pediatric)	Pentanal, heptanal	Oxidative stress [67]	Increased	[67]
CKD (pediatric)	Ethanol	Non-specific, possibly environmental contamination [67]	Increased	[67]

**Table 4:** Overview on studies dealing with e-nose use in CKD.

Sample Population (Cases/Controls)	Disease Studied	e-Nose Technology/Device	Type of Sample (s)	Biomarker (s)	Performances	Reference (s)
62 (42 ESKD, 20 CKD)/11	ESKD, CKD	E-nose equipped with 3 MOS sensors	Body odor	Methane, butane, alcohols, ketones, carbon monoxide, nitrogen oxide, ammonia	ACC: 100% between patients and controls; ACC: 95.2% between ESKD and CKD	[98]
110/335 (117 DM, 110 AIn, 108 HC)	Renal disease	Figaro MOS sensors	Dead-space breath air samples	H_2_, CO, VOCs, H_2_S, CO_2_, NH_3_, NO, NO_2_	SE: 86.57%, SP: 83.47%	[99]
46/20	Different stages of CKD	GNP sensors	Breath samples	Isoprene, acetone, ethylene glycol, acetoin, methylated hydrocarbons, ketones	ACC: 79% between early-stage CKD and controls; ACC: 85% between stage 4 and stage 5 CKD; ACC: 76% between early and advanced CKD	[100]
26/11	ESKD	Nanomaterial-based sensors	Exhaled breath samples	Nonane, methylene chloride, isopropanol, styrene	ACC: 80% with DFA	[101]
40/0	CKD	Figaro TGS2444, MQ135, MQ137, TGS826 MOS sensors	Breath samples	Ammonia	ACC: 88% (for ammonia)	[102]
16/28 (6 DM, 22 HC)	CKD	E-nose equipped with 6 chemical sensors: MQ-2, MQ-3, MQ9, MQ-135, MQ-137 and MQ-138	Breath (and urine) samples	Different VOCs, including ammonia	Correct classification up to 100% with e-nose data analyzed by SVM	[103]
n.a.	Renal diseases (potentially)	E-nose based on PANI nanocomposites	Breath samples simulation	Ammonia	ACC: up to 85% with SVM	[104]
95/n.a.	CKD	FAIMS	Urine headspace	VOC composition	81.4% differentiation of kidney function extremes	[105]
121/0	CKD	V-OSC	Breath samples	Ammonia	ROC AUC = 0.835 (*p* < 0.0001) across CKD at 1st stage and at any stage at 974 ppb (SE: 69%; SP: 95%). ROC AUC = 0.831 (*p* < 0.0001) between patients with/without eGFR < 60 mL/min/1.73 m^2^ (at 1187 ppb: SE: 71%, SP: 78%; at 886 ppb: SE: 80%, SP: 69%)	[106]
51/47	CKD (under hemodialysis)	Figaro MQ135 MOS sensor	Breath samples	Ammonia	ACC: 85.7%	[107]
110/142	Renal cancer	Cyranose 320^®^	Urine sample	Combined VOCs	SP: 89.4%, SE: 71.8%, PPV: 84.04%, NPV: 80.37%; CVA: 81.7%, *p* < 0.001. ROC AUC: 0.85	[108]
6/3	Early and late stage CKD	Fe_2_Mo_3_O_8_/MoO_2_@MoS_2_ nanocomposite gas sensor	Breath samples	Ammonia	R^2^ = 0.99 between ammonia concentrations and sensors’ outputs	[109]

ACC: accuracy; AIn: airway inflammation; AUC: area under the curve; CKD: chronic kidney disease; CVA: cross-validated accuracy; DFA: Discriminant Function Analysis; DM: diabetes; ESKD: end-stage kidney disease; FAIMS: field asymmetric ion mobility spectrometry; GNP: gold nanoparticles; HC: healthy controls; MOS: metal–oxide–semiconductor; n.a.: not available; NPV: negative predictive value; PANI: polyaniline nanocomposites; PPV: positive predictive value; ROC: receiver operating characteristic; SE: sensitivity; SP: specificity; SVM: Support Vector Machine; VOC: volatile organic compounds; V-OSC: vertical-channel organic semiconductor.
